# Estrogen Regulates Ca^2+^ to Promote Mitochondrial Function Through G-Protein-Coupled Estrogen Receptors During Oocyte Maturation

**DOI:** 10.3390/biom14111430

**Published:** 2024-11-11

**Authors:** Qingyang Liu, Jingmei Li, Yanxue Li, Ming Cheng, Hui Zhang, Baohua Ma

**Affiliations:** 1 College of Veterinary Medicine, Northwest A&F University, Yangling 712100, China; lqy511@nwafu.edu.cn (Q.L.); lijingmei1212@163.com (J.L.); 18729232701@163.com (Y.L.); mingcheng984737409@163.com (M.C.); 2 Key Laboratory of Animal Biotechnology, Ministry of Agriculture, Yangling 712100, China

**Keywords:** estrogen, calcium, oocyte maturation, mice

## Abstract

Estrogen is a steroid hormone that plays a key role in regulating many physiological processes, such as follicle activation and development and oocyte maturation in mammals. Ca^2+^ is crucial in oogenesis, oocyte maturation, ovulation, and fertilization. However, the mechanism by which estrogen regulates Ca^2+^ during oocyte maturation in mice has not been reported. This study revealed that Ca^2+^ levels in oocytes significantly increase during the 4–12 h period in vitro. Oocytes treated with 0.1 µM estrogen and 1 µM G1, a G-protein-coupled estrogen receptor (GPER) agonist, showed significantly increased Ca^2+^ levels, while treatment with 1 µM G15, an antagonist of GPER, significantly decreased Ca^2+^ levels. Notably, estrogen regulates Ca^2+^ in oocytes through the GPER pathway and promotes the expression of the Ca^2+^-producing protein EPAC1. In addition, estrogen alleviates the inhibitory effect of the Ca^2+^ chelator BAPTA-AM during oocyte maturation by promoting Ca^2+^ production. Furthermore, estrogen can promote the expression of the mitochondrial generation-associated protein SIRT1 through the GPER pathway, alleviate mitochondrial oxidative damage caused by BAPTA-AM, and restore the mitochondrial membrane potential level. Collectively, this study demonstrates that estrogen can regulate Ca^2+^ through the GPER-EPAC1 pathway and promote the expression of SIRT1, which promotes oocyte mitochondrial function during oocyte maturation.

## 1. Introduction

Follicular development and oocyte maturation regulation are important processes for completing reproductive activity in females. The process of oocyte maturation is divided into nuclear maturation and cytoplasmic maturation. In normally developing oocytes, nuclear maturation is arrested at the first meiotic prophase, allowing primary oocyte meiosis to give the cytoplasm sufficient time to mature, thereby ensuring adequate oocyte growth. Follicular somatic cells transport nutrients and small molecules, such as ions, from the follicle to the oocyte through gap junctions, which contributes to the development and maturation of the oocyte cytoplasm [1]. Ca^2+^ is a widespread intracellular signaling molecule involved in a variety of cellular physiological and biochemical activities [2,3]. Estrogen not only plays an important role in the regulation of estrus and sexual behavior in mammals but also participates in the regulation of primordial follicle activation and development and oocyte maturation. Estrogens exert their physiological effects through receptors, including both nuclear and membrane receptors [4,5]. It was found that the expression of the membrane receptor GPER on mammalian oocyte membranes increased as oocytes matured [6]. Estrogen can induce the production of cGMP by maintaining the expression of Npr2 mRNA in mouse oocytes, and cGMP can further cause the elevation of cAMP [7]. In mouse bone marrow mesenchymal stem cells, cAMP concentration increased after 3–6 h of treatment with G1 but decreased after treatment with G15 [8]. Previous research has found that estrogen can rapidly increase intracellular cAMP via GPER in cumulus cells [9]. By coincidence, increased intracellular cAMP levels lead to the activation of the cAMP effector protein, Epac1, and increase intracellular Ca^2+^ levels via phospholipase C and ryanodine receptor Ca^2+^ release channel. Elevated intracellular Ca^2+^ levels help promote Sirtuin-1(Sirt1) activity, which promotes mitochondrial biosynthesis and improves mitochondrial function [10]. Oocyte maturation is regulated by many factors; however, the mechanism is still unclear. The mitochondrion, as the center of cellular metabolism, is one of the key indicators for controlling the quality of oocytes, which is essential for oocyte maturation.

In this study, we investigated the role of estrogen in regulating Ca^2+^ during oocyte maturation and its related signaling pathways. We explored the possible influence of estrogen on mitochondrial biosynthesis and function through the regulation of Ca^2+^. The findings of this study will contribute to a theoretical basis for understanding the regulation of oocyte maturation, thereby further optimizing the in vitro maturation culture system for mammalian oocytes and facilitating the acquisition of in vitro matured oocytes of good quality and with strong subsequent developmental potential.

## 2. Materials and Methods

### 2.1. Animals and Ethics Statement

Female Kunming mice (6~8 weeks old) were obtained from the Experimental Animal Center of the Xi’an Jiaotong University. They were housed in a temperature (20~25 °C) and light-controlled environment (12 h light/12 h dark cycle) and provided with food and water ad libitum. All experimental protocols and mouse handling procedures were reviewed and approved by the Institutional Animal Care and Use Committee of the College of Veterinary Medicine, Northwest A&F University (No. 2018011212; approved date: 12 January 2018).

### 2.2. Collection and Culture of Mouse Oocytes

Female mice were stimulated by an intraperitoneal injection of 5 IU PMSG (Ningbo Second Hormone Factory), and the mice were sacrificed by cervical dislocation 44 h later. The ovaries were collected, and the well-developed Graafian follicles were punctured with 30-gauge needles to collect oocytes. Only oocytes with morphological integrity and a distinct germinal vesicle (GV) were cultured in basic culture medium consisting of M2 medium at 37 °C in 5% CO_2_ and saturated humidity. In some experiments, the basic culture medium was supplemented with various concentrations of 17β-estradiol (Sigma, St. Louis, MO, United States), G15 (Cayman, Ann Arbor, MI 48108, USA), G1 (Cayman), BAPTA-AM (Selleck, Houston, TX, USA), 1 μM ICI182780 (Cayman, Ann Arbor, MI 48108, USA), and corresponding amounts of dimethyl sulfoxide (DMSO) in controls. All reagent solutions were freshly prepared for each experiment with a final DMSO concentration of no more than 0.1% (V/V).

### 2.3. Ca^2+^ Staining

Mouse oocytes were collected and cultured in vitro with M2 culture medium for 6 h. The oocytes were randomly divided into four groups, incubated with 0.5, 1, 2, and 5 µM Ca^2+^ fluorescent probes (Beyotime, Shanghai, China, S1056) for 30 min at 37 °C under light-avoiding conditions, and then transferred to a special dish for laser confocal observation after being washed three times with the operation solution, or they were incubated with Rhod-2,AM (yeasen, Shanghai, China, 40776ES50) for 30 min at 37 °C under light-avoiding conditions and then transferred to a special dish for laser confocal observation after being washed three times with the operation solution and re-incubated for 20 min. Ca^2+^ staining of the oocytes was observed under a laser confocal microscope (emission 488 nm and 561 nm, 14.5%). The average fluorescence intensity of the oocytes in each group was detected by using Image J 1.52U (NIH, Bethesda, MD, USA).

### 2.4. Detection of Oocyte Mitochondrial Peroxide and Mitochondrial Membrane Potential

Mouse oocytes were collected and randomly divided into four groups: the control group (M2 culture medium containing DMSO), the estrogen-treated group (1 µM), the estrogen co-treated with BAPTA-AM group (1 µM), and the BAPTA-AM-treated group (1 µM). The oocytes were cultured in vitro for 6 h and then incubated for 30 min with either Mito-sox (1:200, Invitrogen, Inc., Carlsbad, CA, USA, M36008) or JC-1 (1:100, Sigma, St. Louis, MO, United States) under light-avoiding conditions, washed three times in M2 media, and then observed and photographed under a fluorescence microscope. The red and green fluorescence levels were measured using confocal microscopy at the same laser power (emission 488 nm and 561 nm, 15.3%). The average fluorescence intensity of different groups was detected using Image J to determine the effect of estrogen on the mitochondrial function of oocytes after BAPTA-AM treatment.

### 2.5. Western Blot Analysis

Mouse oocytes were collected and cultured in vitro for 6 h to extract total protein (at least 60 oocytes per group to ensure that the number of oocytes and the volume of lysate were consistent in each group). Cells were lysed on ice for 30 min using a tissue cell lysate containing 1 mM phenyl methyl sulfonyl fluoride (PMSF, solarbio, Beijing, China) and 1 mM protein phosphatase inhibitor (radio immunoprecipitation assay lysis, RIPA, solarbio, Beijing, China). RIPA was pre-cooled on ice for 10–20 min before sample collection. Oocytes were added and blown several times to accelerate cell lysis. Subsequently, SDS Protein Sampling Buffer was added, mixed well, and incubated in a metal bath at 100 °C for 10 min to fully denature the protein samples. The denatured protein samples (10 μL per well) were loaded into 12% SDS-PAGE gels for vertical electrophoresis. The concentration gel was run at a constant pressure of 80 volts for 45 min, and the separation gel was run at a constant pressure of 110 volts for about 1.5 h. After electrophoresis, the proteins in the SDS-PAGE gel were transferred onto 0.22 μm pore-sized PVDF membranes. The PVDF membranes were activated through soaking in anhydrous methanol for 1 min before transfer, and the membranes were wet-transferred at a constant pressure of 50 V for 2–3 h. After transfer, the membranes were blocked in 5% (*w*/*v*) skimmed milk powder TBST for 1 h at room temperature, followed by incubation with primary antibodies (rabbit anti-GAPDH antibody, 1:2000, proteintech, Cat#60004-1-Ig; rabbit anti-EPAC1 antibody, 1:1000, MCE, Cat#HY-P80120; rabbit anti-GAPDH antibody, 1:1000, MCE, Cat#HY-P80319) at 4 °C overnight. After primary antibody incubation, the membranes were transferred to TBST and washed on a shaker 3 times, 10 min each time, and then incubated with the secondary antibody corresponding to the primary antibody species at room temperature for 2 h. After the membranes had been washed 3 times in TBST, the membrane signals were visualized by using a chemiluminescent HRP substrate reagent (Bio-rad Laboratories, Hercules, CA, USA).

### 2.6. Statistical Analysis

Statistical analyses were performed using GraphPad Prism 8.00 software (GraphPad Software, La Jolla, CA, USA). The data are reported as means ± SEM. The results of statistically significant differences are denoted by asterisks (* *p* < 0.05, ** *p* < 0.01, *** *p* < 0.001, and **** *p* < 0.0001).

## 3. Results

### 3.1. Estrogen Affects Ca^2+^ Levels in Mouse Oocytes

**Figure 1:**
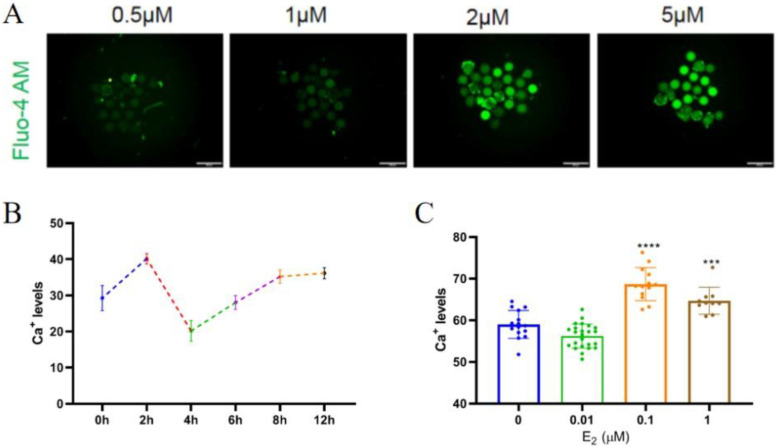
Estrogen affects Ca^2+^ levels in mouse oocytes. (**A**) The staining results of different concentrations of Ca^2+^ fluorescent probes. From left to right, the results of Ca^2+^ fluorescence probe staining at 0.5, 1, 2, and 5 µM are shown. Scale bar, 100 μm. (**B**) The changes in Ca^2+^ concentration during in vitro culture of mouse oocytes. (**C**) The effect of different concentrations of estrogen on Ca^2+^ levels in oocytes. The bars indicate the mean ± SEM of at least 20 oocytes. *t*-test for analysis of variance: *** *p* < 0.001 and **** *p* < 0.0001.

To investigate whether estrogen affects Ca^2+^ levels in mouse oocytes, mouse oocytes were stained with different concentrations of the Ca^2+^ fluorescent probe Fluo3-AM. It was found that 2 µM was the optimal concentration of Fluo3-AM for staining mouse oocytes (Figure 1A); thus, 2 µM Fluo3-AM was chosen for subsequent experiments to detect Ca^2+^ fluorescence of mouse oocytes. While exploring the optimal time for culturing mouse oocytes in vitro, it was observed that there were two fluctuations in oocytes during the in vitro culture process. The lowest Ca^2+^ level was observed at 4 h of in vitro culture, followed by a significant increase in Ca^2+^ levels from 4 to 12 h of culture (Figure 1B). Notably, the treatment of mouse oocytes with different concentrations of estrogen for 6 h revealed that 0.1 and 1 µM of estrogen highly significantly increased Ca^2+^ levels (*p* < 0.001) (Figure 1C). Hence, 1 μM was applied in subsequent experiments.

### 3.2. Estrogen Affects Mouse Oocyte Ca^2+^ Levels via Membrane Receptors

**Figure 2:**
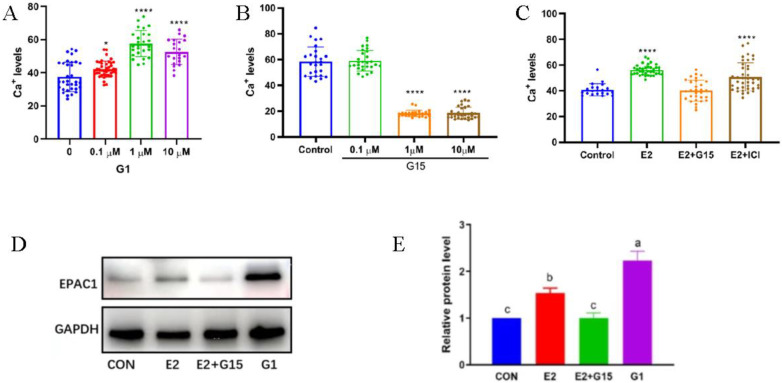
Estrogen affects mouse oocyte Ca^2+^ levels via membrane receptors. (**A**) The effect of different concentrations of G1 on Ca^2+^ levels in oocytes. The bars indicate the mean ± SEM of at least 20 oocytes. *t*-test for analysis of variance: * *p* < 0.05, **** *p* < 0.0001. (**B**) The effect of different concentrations of G15 on Ca^2+^ levels in oocytes. The bars indicate the mean ± SEM of at least 20 oocytes. *t*-test for analysis of variance: **** *p* < 0.0001. (**C**) The results of estrogen promotion of oocyte Ca^2+^ levels via the membrane receptor pathway. The bars indicate the mean ± SEM of at least 20 oocytes. *t*-test for analysis of variance: **** *p* < 0.0001. (**D**) The representative plots of EPAC1 and the internal reference protein GAPDH, and the grayscale analysis is shown in (**E**); column height and the error line indicate the mean ± SEM of the grayscale analysis test results. Different letters indicate significant differences (*p* < 0.05).

To investigate the effect of G1 on Ca^2+^ concentration in oocytes, mouse oocytes were treated with different concentrations of G1. It was found that 1 µM G1 significantly increased Ca^2+^ levels in mouse oocytes (*p* < 0.0001) (Figure 2A). To investigate the effect of G15 on Ca^2+^ levels in oocytes, mouse oocytes were treated with different concentrations of G15. It was found that 1µM G15 highly significantly down-regulated Ca^2+^ levels (*p* < 0.0001) (Figure 2B). Mouse oocytes were treated with estrogen, estrogen combined with G15, and the nuclear receptor inhibitor ICI. It was found that estrogen significantly promoted the Ca^2+^ levels of oocytes, and the group co-treated with estrogen and ICI at the same concentration also had significantly promoted Ca^2+^ levels of oocytes, indicating that ICI could not inhibit the regulatory effect of estrogen on the Ca^2+^ levels of mouse oocytes. In contrast, in the G15 and estrogen co-treatment group, the Ca^2+^ levels in oocytes were not significantly different from those in the control group. This suggests that estrogen significantly promoted oocyte Ca^2+^ levels through the membrane receptor (Figure 2C). Mouse oocytes were treated with estrogen, estrogen combined with G15, and G1 and assayed by Western blot analysis. It was found that both estrogen and G1 treatments promoted EPAC1 protein expression, whereas G15 inhibited the promotion of EPAC1 by estrogen, suggesting that estrogen promotes the expression of EPAC1 through the GPER pathway (Figure 2D,E).

### 3.3. Estrogen Alleviates the Inhibitory Effect of BAPTA-AM on Oocyte Maturation in Mice

**Figure 3:**
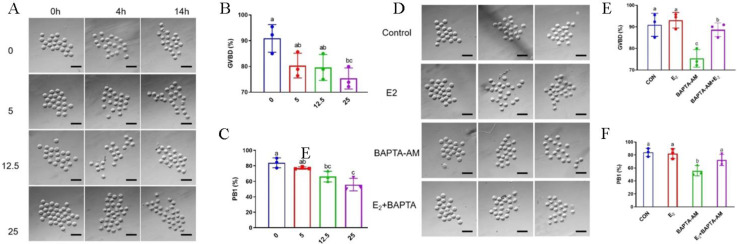
Estrogen alleviates the inhibitory effect of BAPTA-AM on oocyte maturation in mice. (**A**) A representation of the effect of BAPTA-AM treatment at different concentrations (0, 5, 12.5, 25 µM) on oocytes treated for 4 h and 14 h. Scale bar, 100 μm. (**B**,**C**) GVBD and PB1 rates. Column height and the error line indicate the mean ± SEM of the results from 3 tests; different letters indicate significant differences (*p* < 0.05). (**D**) Oocytes were treated with estrogen, BAPTA-AM combined with estrogen, and BAPTA-AM for 4 h and 14 h. Scale bar, 100 μm. (**E**,**F**) GVBD and PB1 rates; column height and the error line indicate the mean ± SEM of the results from 3 tests. Different letters indicate significant differences (*p* < 0.05).

In order to explore the effect of estrogen-regulated Ca^2+^ production on oocyte maturation, the effect of BAPTA-AM on oocyte maturation in mice was first analyzed. Mouse oocytes were treated with different concentrations of BAPTA-AM. GV-stage oocytes were isolated (0 h) and cultured for 4 h and 14 h, and the rates of germinal vesicle breakdown (GVBD) and first polar body (PB1) extrusion were counted. The rate of GVBD and PB1 extrusion was significantly inhibited by BAPTA-AM in a dose-dependent manner, and the rates of GVBD and PB1 were significantly down-regulated by the treatment of 25 µM BAPTA-AM (*p* < 0.05), suggesting an inhibitory effect of Ca^2+^ chelators on oocyte maturation (Figure 3A–C). To further analyze whether estrogen could alleviate the inhibitory effect of BAPTA-AM on oocyte maturation, oocytes were treated with estrogen, BAPTA-AM combined with estrogen, and BAPTA-AM. Isolated GV-stage oocytes (0 h) were photographed and analyzed for GVBD rate and PB1 rate. BAPTA-AM treatment inhibited the GVBD and expulsion of PB1, whereas estrogen alleviated the inhibitory effect of BAPTA-AM on the maturation of oocytes, which indicates that estrogen alleviated the inhibitory effect of Ca^2+^ chelators on the maturation of oocytes through the promotion of Ca^2+^ generation (Figure 3D–F).

### 3.4. Estrogen Alleviates the Inhibitory Effect of BAPTA-AM on Mitochondrial Function in Mouse Oocytes

**Figure 4:**
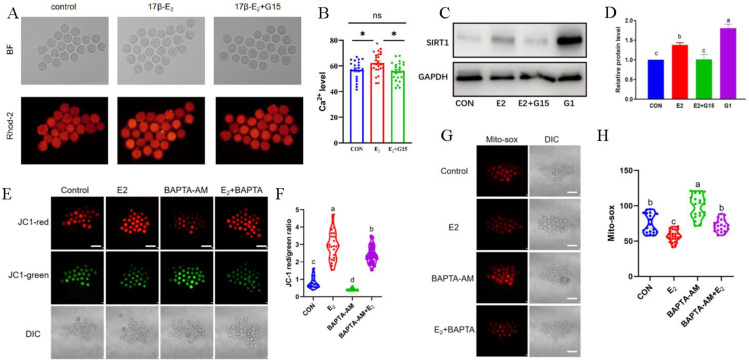
Estrogen alleviates the inhibitory effect of BAPTA-AM on mitochondrial function in mouse oocytes. (**A**) The Rhod-2 staining results of oocytes cultured in vitro for 6 h in different treatment groups. (**B**) Fluorescence intensity analysis. The bars indicate the mean ± SEM of at least 20 oocytes. *t*-test for analysis of variance: ns *p* > 0.05, * *p* < 0.05. Scale bar, 200 μm. (**C**) The representative plots of SIRT1 and GAPDH (Whole Western blot from Figure 2 and Figure 4 can be found in supplementary materials sections). (**D**) Grayscale analysis: column height and the error lines indicate the mean ± SEM of the grayscale analysis test results; different letters indicate significant differences (*p* < 0.05). (**E**) The JC1 staining results of oocytes cultured in vitro for 6 h in different treatment groups. (**F**) Red/green fluorescence intensity ratio (mitochondrial membrane potential) analysis: column height and the error lines represent the mean ± SEM of at least 20 oocytes; different letters indicate significant differences (*p* < 0.05). Scale bar, 200 μm. (**G**) The Mito-sox staining results of oocytes cultured in vitro for 6 h in different treatment groups. (**H**) Fluorescence intensity analysis: column height and the error lines represent the mean ± SEM of at least 20 oocytes; different letters indicate significant differences (*p* < 0.05). Scale bar, 200 μm.

To investigate the effect of estrogen regulation of Ca^2+^ production on mitochondrial function in oocytes, treatments with estrogen and a combination of estrogen and G15 were applied. After 6 h of in vitro culture, it was found that compared to the control group, the mitochondrial Ca^2+^ levels in oocytes were significantly increased in the estrogen treatment group. The mitochondrial Ca^2+^ levels in the estrogen group were extremely significantly elevated when compared with those of the group co-treated with estrogen and G15. This demonstrates that estrogen affects mitochondrial Ca^2+^ levels in mouse oocytes through the GPER pathway (Figure 4A,B). Mouse oocytes were treated with estrogen, estrogen combined with G15, and G1. Using Western blot analysis, we found that both estrogen and G1 treatments promoted SIRT1 protein expression, whereas G15 inhibited the promotion of SIRT1 expression by estrogen, suggesting that estrogen promotes Ca^2+^ levels through the GPER pathway and promotes the expression of SIRT1, a key protein downstream of the Ca^2+^ signaling pathway (Figure 4C,D). To investigate the effect of BAPTA-AM on mouse oocytes, mouse oocytes were divided into the control group, the estrogen-treated group, the BAPTA-AM-treated group, and the estrogen co-treated with BAPTA-AM group, all treated for 6 h. Oocytes cultured in vitro for 6 h were stained with Mito-sox and JC1. We found that BAPTA-AM treatment significantly increased the mitochondrial superoxide level, suggesting that the reduction in Ca^2+^ can cause mitochondrial damage. However, estrogen treatment significantly down-regulated the mitochondrial superoxide levels in oocytes, indicating that estrogen alleviated the mitochondrial oxidative damage caused by BAPTA-AM (Figure 4E,F). Furthermore, BAPTA-AM treatment significantly down-regulated the mitochondrial membrane potential level, suggesting that the reduction in Ca^2+^ causes the inhibition of mitochondrial function. In contrast, estrogen treatment significantly increased the mitochondrial membrane potential level in oocytes. These results suggest that estrogen alleviates the impairment of mitochondrial function in oocytes caused by BAPTA-AM through Ca^2+^ promotion (Figure 4G,H).

## 4. Discussion

Ca^2+^ is one of the major signaling molecules that plays a central role in meiotic resumption in oocytes. A moderate rise in Ca^2+^ within the physiological range leads to meiotic resumption through a mechanism by which NPPC binds to its receptor NPR2, producing cGMP that maintains the meiotic block. However, the LH-dependent EGF receptor pathway elevates the concentration of Ca^2+^. High levels of Ca^2+^ reduce the affinity of NPP2 for NPPC, which in turn leads to the decline in cGMP and meiotic resumption [11]. Estrogen is the major steroidal sex hormone, formed by androgen demethylation [12] and originally found in the uterus [13]; it is produced primarily by the ovaries, adipose tissue, and adrenal cortex. Estrogen is divided into three categories: estradiol, estriol, and estrone. However, because estrone levels are higher during menopause and estriol plays more of a role during pregnancy, the estrogens being explored generally refer to estradiol. Estradiol is mainly synthesized and secreted in the ovaries by follicular granulosa cells and the corpus luteum [14]. Estrogen exerts a variety of physiological effects by acting on different receptors, which are mainly categorized into two main groups: nuclear and membrane receptors. GPER plays an important role in mediating the process of meiotic blockage maintained by estrogen [15]. Preliminary studies in our laboratory have shown that GPER is expressed at all stages of mouse oocytes, and it has been shown that signaling pathways mediate the involvement of GPER in the regulation of oocyte maternal mRNA translation, affecting oocyte maturation and embryo development. In dormant mouse blastocysts, estrogen may induce an increase in intracellular Ca^2+^ by acting on the composition of the cell membrane [16]. In mouse sperm, the endogenous GPER ligand 17β-estradiol and the selective G1 increase intracellular Ca^2+^ concentration, which can be abolished by G15 [17]. This study found that ICI (nuclear receptor inhibitor) did not inhibit the estrogen effect, while G15 inhibited the estrogen effect, indicating that estrogen regulates Ca^2+^ through the GPER pathway. Subsequently, it was further verified by WB that G1 promotes EPAC1 production, while G15 inhibits estrogen. It has been shown that estrogen regulates Ca^2+^ through the GPER pathway. Considering the scarcity of oocyte samples, G15 is not added to every experiment in this study. Additionally, it has been demonstrated that estrogen can cause elevated cAMP and Ca^2+^ concentrations through multiple ways [18,19]. EPAC1 is a target of cAMP signaling and a regulator of Ca^2+^ signaling [20]. In melanoma cells, EPAC1 increases intracellular calcium content. Specifically, Epac1 can increase Ca^2+^ in the endoplasmic reticulum through the IP3 receptor [21,22], while the induced elevation of Ca^2+^ in the extracellular space inhibits the Epac1-induced release of Ca^2+^ from the ER [23]. Therefore, we suggested that elevated intracellular calcium is not related to extracellular calcium, but further investigation is still needed. In HeLa cells, it was found that cAMP in mitochondria affects mitochondrial Ca^2+^ levels through EPAC1 [24]. Moreover, EPAC1 facilitates the transfer of calcium from the endoplasmic reticulum (ER) to the mitochondria through a macromolecular complex. This complex is composed of voltage-dependent anion channel 1 (VDAC1), chaperone glucose-regulated protein 75 (GRP75), inositol 1,4,5-trisphosphate (IP3) receptor 1 (IP3R1), and mitochondrial Ca^2+^ uniporter (MCU) [25]. In cardiomyocytes, EPAC1 acts by stimulating Ca^2+^ exchange between the ER and mitochondria [26]. EPAC1 has not been found to be activated in a cAMP-independent manner, but it has been suggested that the inhibition of EPAC1 reduces Ca^2+^ uptake by mitochondria [27]. In addition, the intracellular Ca^2+^ concentration in vascular smooth muscle cells of Epac1+/+ mice is significantly increased, while the elevation of Epac1+/+ is significantly attenuated [28]. Therefore, we suggested that Ca^2+^ in oocytes might be regulated through the estrogen–GPER-EPAC1 pathway. This study demonstrates that estrogen leads to an increase in EPAC1 in mouse oocytes. In other words, estrogen can induce Ca^2+^ production through cAMP, which is consistent with previous research findings [10]. However, ERα and GPER are also regulated by Ca^2+^ at the receptor level, which means downstream signaling is regulated through feed-forward loops [29]. Therefore, it is also hypothesized that Ca^2+^ can reduce intracytoplasmic cAMP levels in turn. In mammalian oocytes, Ca^2+^ signaling represents a vital mechanism [30]. Although the role of Ca^2+^ in GVBD remains controversial, microinjection of Ca^2+^ chelators inhibits polar body abscission in Xenopus laevis oocytes and promotes the formation of PB1 when Ca^2+^ concentrations are elevated [31]. In this experiment, we found that the lack of Ca^2+^ significantly decreased the GVBD and PB1 rates of mouse oocytes during in vitro development. This decrease was alleviated by the addition of estrogen, suggesting that BAPTA-AM inhibits the maturation of mouse oocytes and that estrogen can counteract this inhibition by promoting Ca^2+^ production. Sirt1 is the most prominent studied member of sirtuins, and its activity can significantly impact mitochondrial function, such as regulating mitochondrial biogenesis and turnover [32].

The Sirt1 activator SRT1720 has shown positive effects on female reproduction, including increasing the follicle reserve and improving ovarian lifespan. Additionally, it plays a significant role in regulating inflammation and mammalian metabolism and delaying aging, and is important in the study of oocyte quality [33]. In Sirt1-deleted mice, the absence of Sirt1 in oocytes of mice aged 9–11 months led to a decrease in fertility; the average number of pups decreased overall, and 50% of cases had a complete loss of fertility. The deletion of oocyte Sirt1 also resulted in a significant delay in bipolar spindle assembly and chromosomal arrangement, and Sirt1-deficient oocytes have been shown to impair preimplantation embryonic development through increased oxidative stress [34]. Oocytes undergo a complex process prior to ovulation, during which most of the energy required is derived from mitochondrial ATP. Additionally, mitochondria, as centers of cellular metabolism, are important for oocytes because of their ability to influence gene expression. It has been shown that SIRT1 and its substrate PGC1α can directly influence mitochondrial transcription, but their exact functions are controversial, and the extent of their effects varies across different cellular environments [35]. Estrogen treatment has been found to up-regulate the expression of SIRT1 in various cell types [36,37]. The overexpression of the sirtuin family member Sirt2 enhances mitochondrial membrane potential and ATP levels, thereby improving mitochondrial function [38]. This study found that estrogen can up-regulate the expression of the mitochondrial biogenesis protein SIRT1 in mouse oocytes by inducing Ca^2+^ production. When mouse oocytes were treated with BAPTA-AM in this experiment, it was found that mitochondrial superoxide levels significantly increased, while mitochondrial membrane potential levels significantly decreased. However, co-treatment with estrogen and BAPTA-AM revealed that estrogen significantly down-regulated mitochondrial superoxide levels and significantly increased mitochondrial membrane potential levels in mouse oocytes. These results suggest that estrogen alleviates the detrimental effects of BAPTA-AM on mitochondrial function in mouse oocytes by promoting Ca^2+^ production. This indicates that estrogen regulation of Ca^2+^ levels may affect mitochondrial function through its impact on the mitochondrial protein SIRT1.

Collectively, our studies demonstrated that estrogen could regulate Ca^2+^ levels through the GPER-EPAC1 pathway, promoting SIRT1, which enhances mitochondrial function in oocytes and subsequently promotes oocyte maturation. Furthermore, research indicates that SIRT1 activation significantly inhibits reactive oxygen species (ROS) production, thereby alleviating oxidative damage [39].

Additionally, SIRT1 participates in spindle formation during meiosis by altering histone acetylation levels [40]. This suggests that during oocyte maturation, estrogen may reduce intracellular oxidative damage by regulating Ca^2+^ levels, promoting correct spindle assembly, enhancing mitochondrial biogenesis and function, and thereby improving the developmental potential of oocytes. However, further research is needed to elucidate the regulatory mechanisms of mammalian oocyte maturation in vitro, essential for refining culture systems and yielding oocytes with robust developmental potential. This study also provides theoretical support for advancing livestock embryo engineering technologies, such as IVF, microfertilization, somatic cell cloning, and gene editing for disease resistance and trait enhancement.

## Data Availability

The original contributions presented in this study are included in the article. Further inquiries can be directed to the corresponding author(s).

## Supplementary Materials

The following supporting information can be downloaded at: https://www.mdpi.com/article/10.3390/biom14111430/s1.
