# Supplementary figures and images for: Estrogen Regulates Ca2+ to Promote Mitochondrial Function Through G-Protein-Coupled Estrogen Receptors During Oocyte Maturation
Source: Biomolecules. 2024 Nov 11;14(11):1430. doi: 10.3390/biom14111430 (PMC11591592; doi:10.3390/biom14111430)

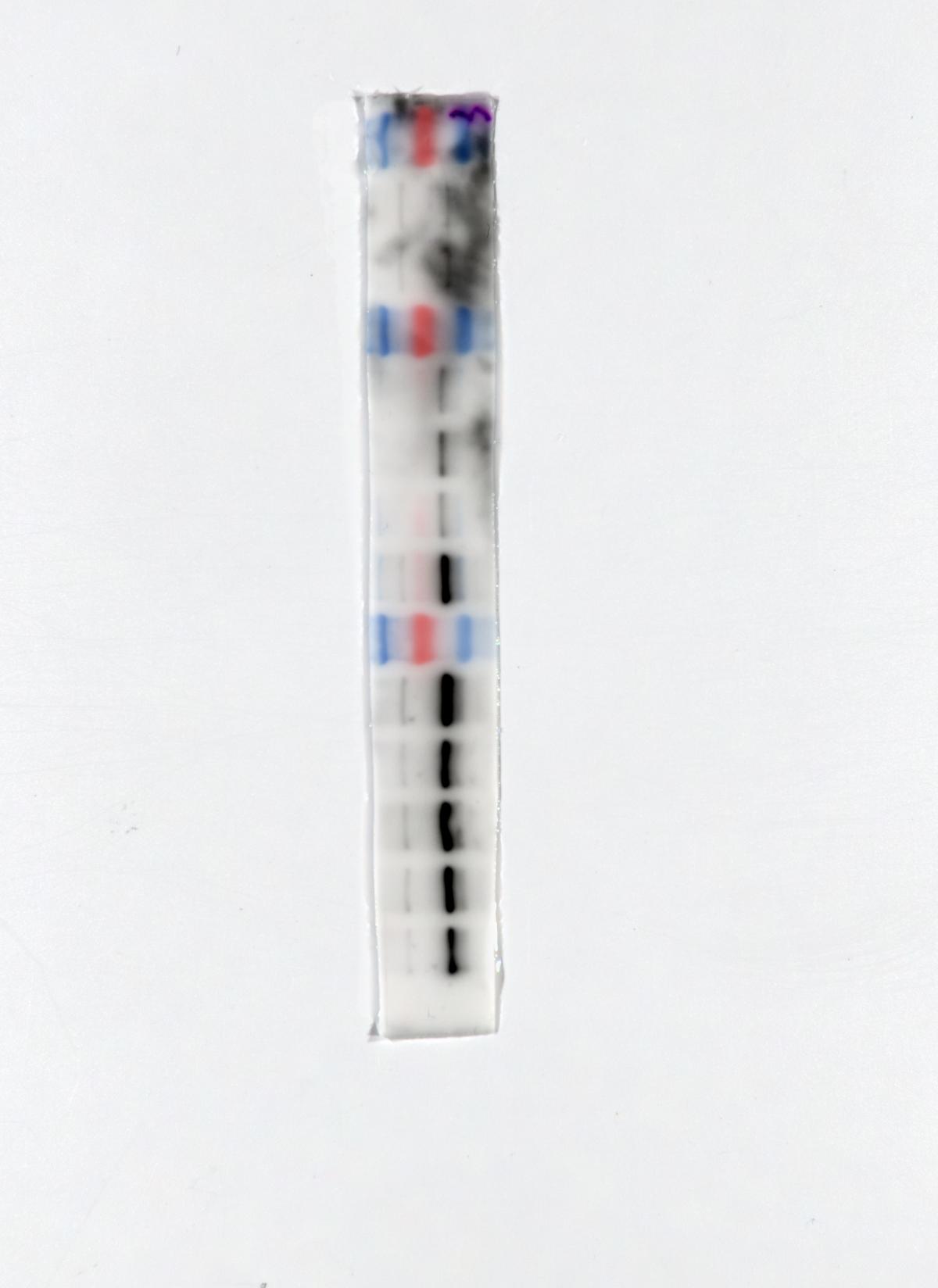

Supplement: Supplementary file 1 [file biomolecules-14-01430-s001.zip › blots/Figure 2D EPAC1.jpg]

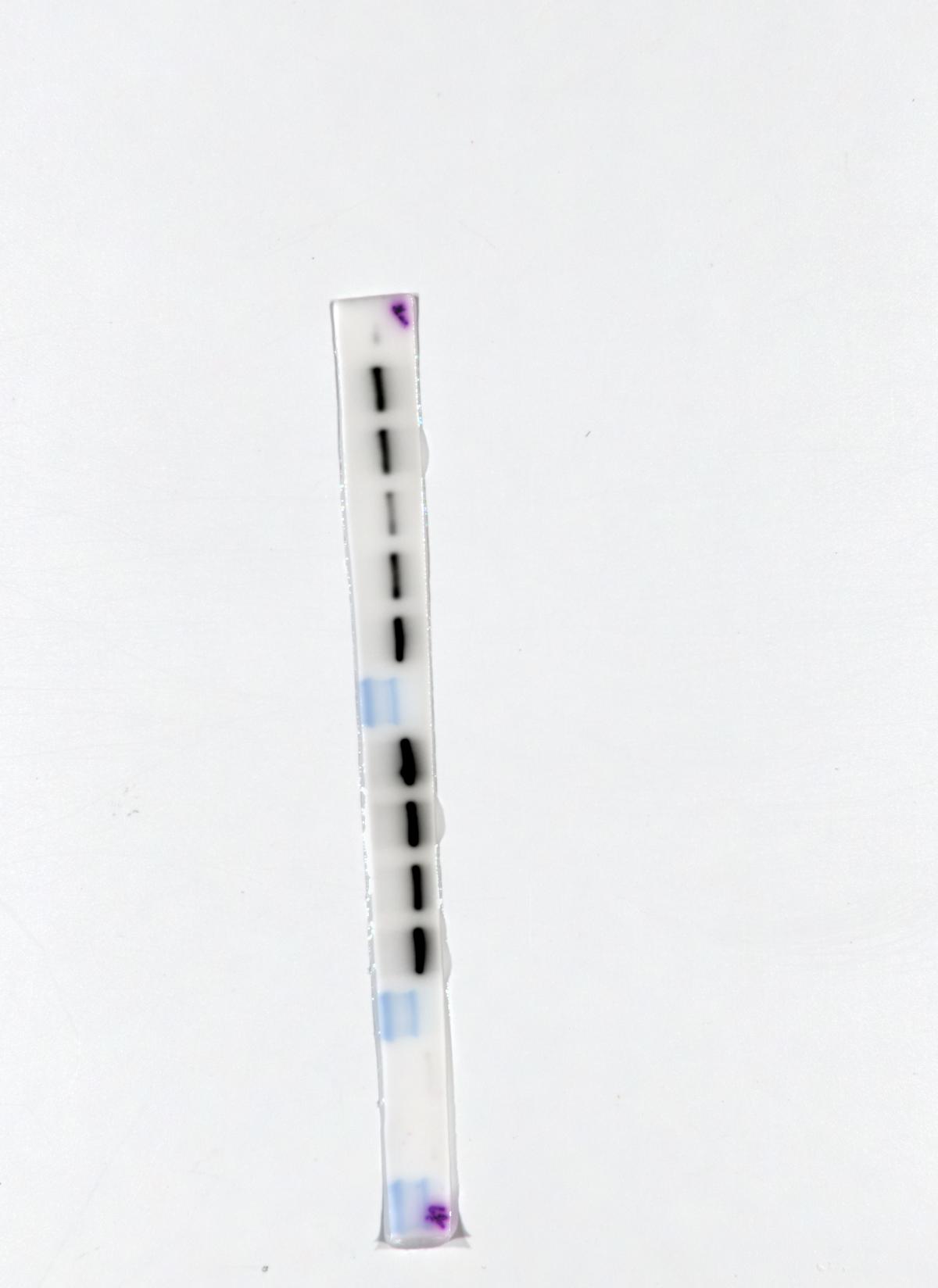

Supplement: Supplementary file 1 [file biomolecules-14-01430-s001.zip › blots/Figure 2D GAPDH.jpg]

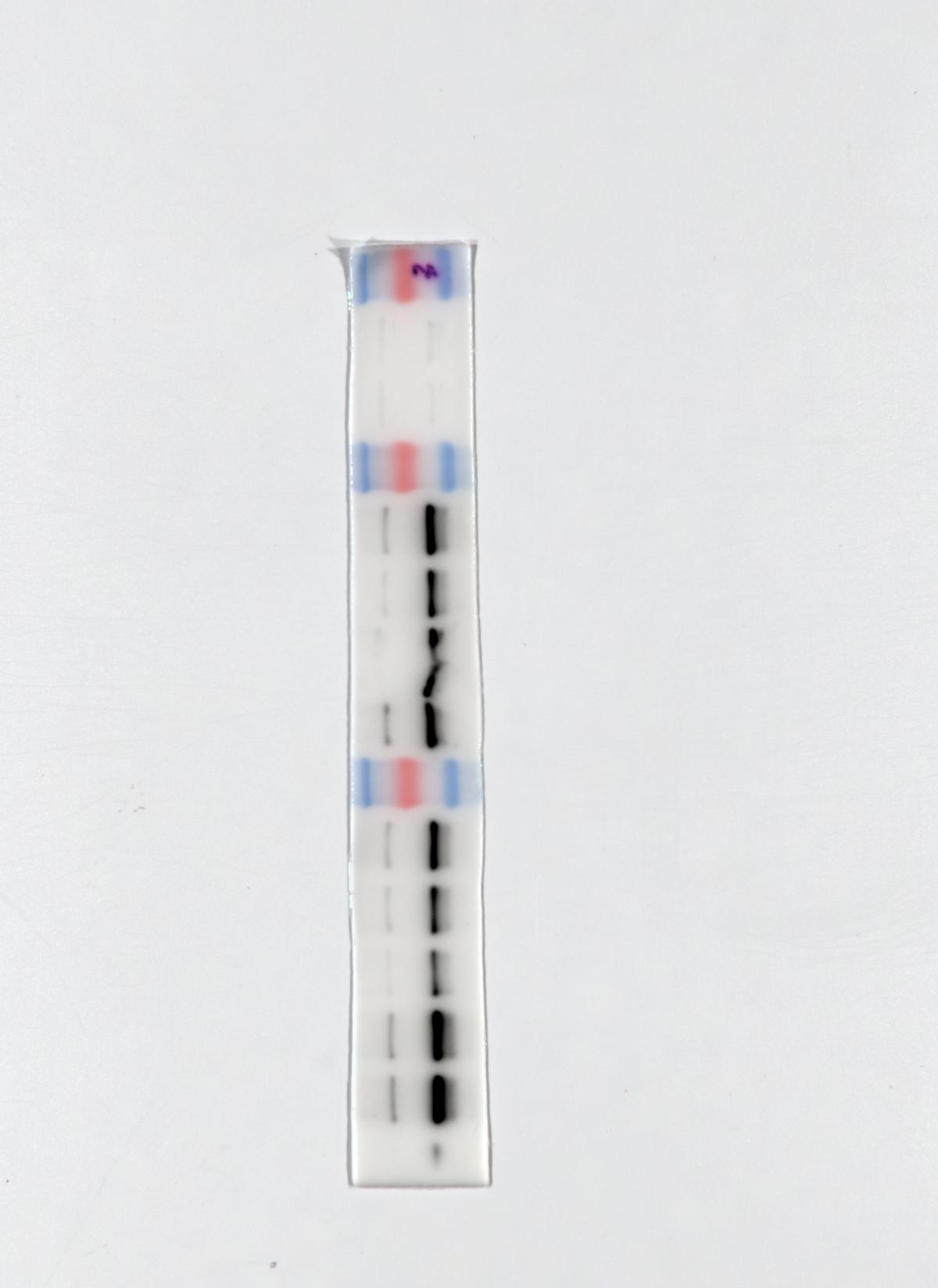

Supplement: Supplementary file 1 [file biomolecules-14-01430-s001.zip › blots/Figure 4 C GAPDH.jpg]

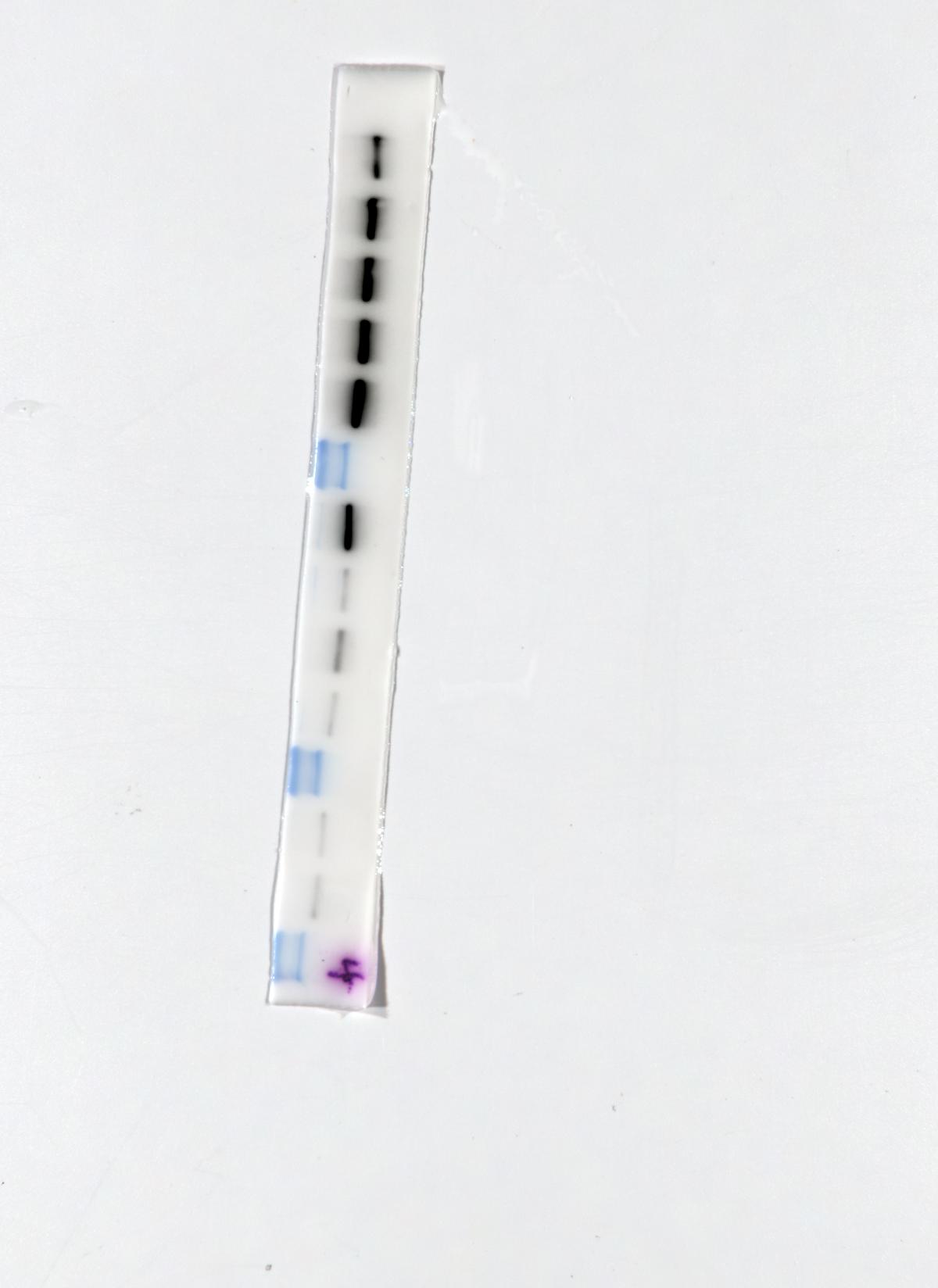

Supplement: Supplementary file 1 [file biomolecules-14-01430-s001.zip › blots/Figure 4 C sirt1.jpg]
